# Optimizing tensile strength and energy consumption for FDM through Mixed-Integer Nonlinear Multi-objective optimization and design of experiments

**DOI:** 10.1016/j.heliyon.2024.e30164

**Published:** 2024-04-23

**Authors:** Saleem Ramadan, Qutaiba Altwarah, Mohammad Abu-Shams, Duha Alkurdi

**Affiliations:** a Industrial Engineering Department, School of Engineering Technology, Al Hussein Technical University, Amman, 11831, Jordan; b School of Engineering and Technology, Central Michigan University, Mt. Pleasant, MI, 48859, USA; c Industrial Engineering Department, School of Applied Technical Sciences, German Jordanian University, Amman, 11180, Jordan; d Industrial and Manufacturing Systems Engineering, University of Michigan at Dearborn, Dearborn, MI, 48128, USA

**Keywords:** Additive manufacturing, Design of experiments, Taguchi design, Response surface design, Nonlinear Multi-objective optimization, 3D printing, FDM

## Abstract

This study presents a methodology for optimizing key parameters of a fused deposition modeling (FDM) printer to minimize energy consumption (EC) while exceeding a specified tensile strength (TS) threshold. Employing Design of Experiments (DoE) with Taguchi and Response Surface analysis, we identify influential parameters affecting TS and EC. A Mixed-Integer Nonlinear Multi-Objective Optimization model is then utilized to balance TS and EC, resulting in optimal parameter values. Validation using fabricated specimens demonstrates less than 5 % error in Tensile Strength and less than 2 % error in Energy Consumption, confirming the efficacy of the proposed methodology.

## 1 Introduction

Three-dimensional (3D) additive manufacturing technology has gained significant use in recent years across diverse fields like food, healthcare, manufacturing, architecture, biomedical, aerospace, and education [1]. Within the medical domain, 3D printing has proven instrumental in creating personalized medical equipment, implants, and surgical instruments [[2], [3], [4]]. For instance, it has been utilized in bone grafting, preparing bone tissue scaffolds, and even for organ transplants [5,6]. In the food industry, 3D printing has been employed to craft personalized meals tailored to individual caloric needs [7,8]. The architectural sector has benefited from 3D printing by enabling the creation of intricate building models, aiding architects in visualizing and testing their designs prior to actual construction [9]. Over the last few years, 3D printing technology has become a vital component in adhering to good manufacturing and production practices. The adoption of 3D printing brings about distinct advantages such as the ability to produce non-standard and complex shapes, cost-effective and rapid production of replacement parts, personalization of products according to specific needs, elimination of tool production, and reduced energy consumption [10]. In the biomedical industry, where unique and specialized products are often required, 3D printing is used to efficiently produce a smaller quantity of customized end-user elements at a reasonable cost and within a shorter time frame compared to traditional manufacturing processes [11]. In orthodontics, 3D printing is employed to design and print customized orthodontic brackets during patient treatment, providing accurate and complex brackets swiftly to patients using a desktop 3D printer and suitable material [12]. Similarly, custom respirators are manufactured using high-precision 3D printers to produce molds for casting silicone masks, minimizing tool production usage [13].

Energy consumption and CO2 emissions from various manufacturing processes pose significant environmental concerns. Energy consumption from traditional manufacturing processes has been on the rise in recent decades. Alternative manufacturing methods, such as 3D printing, offer a solution by reducing energy consumption and carbon footprint across various manufacturing practices [14]. The use of 3D printing minimizes material waste and energy consumption by using only the necessary materials, eliminating tooling requirements, reducing the number of production steps, allowing for local production of parts and products, and decreasing the need for long-distance transportation [15,16]. Moreover, 3D printing technology provides design flexibility and offers various techniques to create structures and products, all controlled by setting parameters. Altering these parameters affects the mechanical and structural properties of the designated product, influencing qualities such as strength, accuracy, shape, and quality [[17], [18], [19]].

Optimization is widely used in all engineering applications and it plays a crucial role in enhancing efficiency, safety, minimizing costs, and improving decision-making across. It allows us to make better use of resources, time, and energy to achieve desired outcomes in an optimal manner. There are various engineering methods and statistical tools used for optimization across different engineering fields. In structural design, researchers [[20], [21], [22], [23]] studied the damage assessment for different composite beams by using different dynamic parameters. The effect of depth of crack and location, initial cracks at different positions, and fiber orientation and lamina stacking sequence on dynamic parameters of the hybrid beam were all studied and analyzed. For example, Taguchi L_27_ design with varying machining conditions such as spindle speed, axial feed rate, and depth of cut were used in machining FG specimens. The response surface method and genetic algorithm were used to determine the optimal machining limit value [24]. In the field of manufacturing processes and machining of hardened materials, Jena et al. [[25], [26], [27]] used various modeling and optimization techniques to obtain the optimal process parameter for machining. In all cases, optimal cutting conditions with using suitable process parameters used to minimize the surface roughness and improve surface quality. For instance, Mahapatra et al. [28] investigated the tool vibration, surface roughness, and chip formation using a response surface method and Gilbert's machining economic model for a specimen of hot work AISI H13. They developed a new-generation of AlTiSiN coated carbide cutting tools that utilized for machining of hot work tool steel. On the other hand, Pradhan et al. [29] used a computational fluid dynamics technique along with an experimental approach to study the effect of machining constraints on target surface erosion rate and surface roughness using HAJMing. They concluded that the function of the machining constrains are the generated workpiece surface contour and erosion rate. Kumar et al. [30] improved the structural integrity for the printed AlSi10Mg parts by using Laser Powder Bed Fusion (LPBF) technique. By attaining an effective melt pool formation, they eliminated the balling and sparring effects, keyhole and cavity formation. LPBF parameters are modeled using Complex Proportional Assessment to optimize surface attributes and mitigate defects like roughness, hardness, and porosity. Similarly, Murugesan et al. [31] used a correlation of LPBF process parameters such as laser power, scan speed, and hatching distance to achieve defect-free Cu–Cr–Zr parts. Defect formations such as balling effects, spattering effects, and un-melted powders could be reduced by analyzing and evaluating the interactions of parameters.

Researchers have investigated the optimization of 3D printing parameters using different testing techniques. Gohil et al. [32] tested a series of different specimens with a 365 nm wavelength photopolymer resin. The printing time, layer thickness, and layer exposure time reviled that their designed and fabricated DPL 3D printer is a low in cost with good accuracy. Hamilton et al. [33] optimized microstructure joints' performance through experimental testing and finite element analysis in 3D printing. Their results demonstrated the statistical significance of all process parameters, with each contributing differently to the response variables. Antonara et al. [34] studied the impact of different process parameters on the production of polymeric microneedles in 3D printers. In their experiments, they identified path width and printing speed as the most significant parameters in the first trial and the percentage of PVA and loading dose as the most significant parameters in the second trial. Griffiths et al. [35] examined the optimization of energy and waste during 3D printing part production. They found that the slice orientation parameter significantly affected scrap weight, while infill percentage and layer height were significant factors for mean part weight and energy consumption/mean production time, respectively. Eguren et al. [36] studied the effect of process parameters on various response variables during an additive 3D printing process. They concluded that extrusion width was the most significant factor based on the normal plots and pareto charts. Kumar et al. [37] investigated the flexural strength and ultimate tensile strength of MEX processed PLA. Using the Taguchi-CRITIC embedded WASPAS approach, they reached an optimal solution for parameters to achieve the maximum mechanical strength, including both flexural and tensile strength. Zaman et al. [38] utilized the Taguchi design to study the impact of fused deposition modeling on the strength of built parts by 3D printers. Their results highlighted the infill percentage as the most significant factor, followed by shells, layer thickness, and infill pattern.

This manuscript addresses a significant research gap in the current literature by focusing on the simultaneous optimization of Fused Deposition Modeling (FDM) 3D printer parameters for conflicting objectives, specifically minimizing energy consumption while maximizing tensile strength. While prior studies recognize the importance of 3D printing across industries, they often concentrate on individual parameter optimization or specific applications. To address this, our study employs a Design of Experiments (DoE) approach to comprehensively tackle the trade-off between energy consumption and tensile strength in FDM technology.

Our investigation examines into eight crucial parameters associated with FDM 3D printing: Infill Percentage, Layer Height, Number of Walls, Nozzle Temperature, Printing Speed, Plate Temperature, Number of Bottom and Upper Layers, and Infill Pattern. Using a fractional design of experiment during a screening phase, we identify the most influential parameters within this initial set of eight. Subsequently, we conduct a Response Surface analysis to establish equations linking Energy Consumption and Tensile Strength to these influential parameters.

**Fig. 1:**
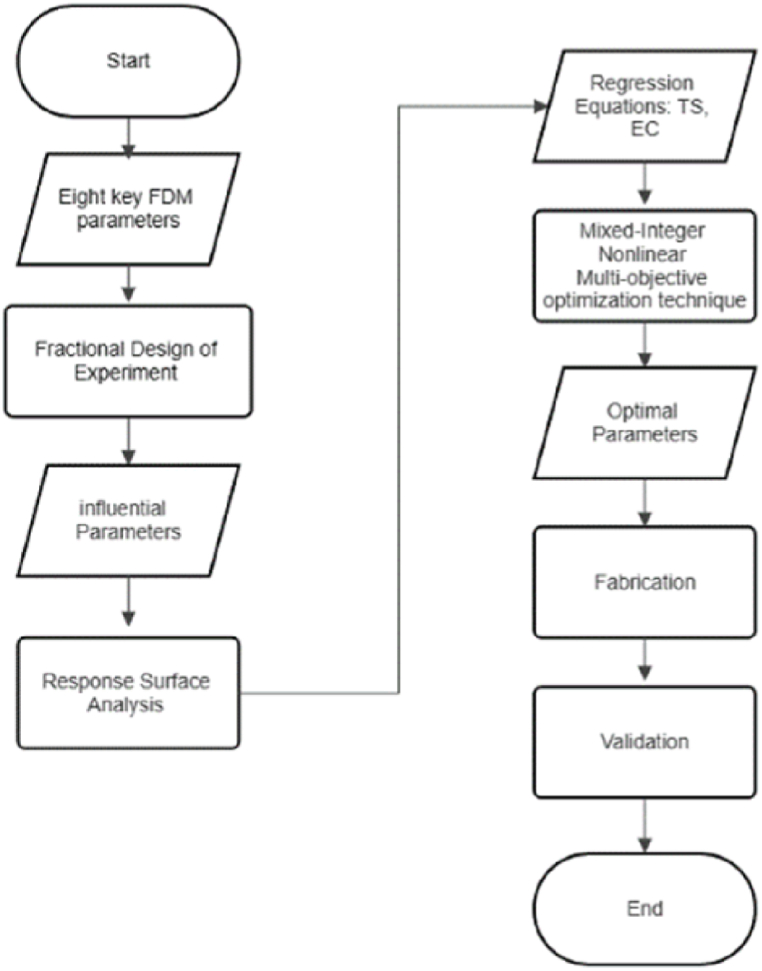
Flowchart for the proposed method.

This study introduces a novel dimension to the existing body of research by applying a Mixed-Integer Nonlinear Multi-objective optimization technique. Emphasizing the need for a more integrated and environmentally conscious optimization strategy in 3D printing, the methodology ensures a holistic approach. The optimal parameter values obtained through this approach effectively balance the conflicting goals of minimizing Energy Consumption and maximizing Tensile Strength. To validate these optimal values, a new set of specimens is fabricated, and the actual Tensile Strength (TS) and Energy Consumption (EC) results are compared with the predicted outcomes. The methodology flow is illustrated in Fig. 1, highlighting the practical and innovative application of the proposed approach.

The remainder of the paper is organized as follows: In Section 2, we outline the design of the specimen and the machine setup. Additionally, we provide an overview of the design of experiments for the screening phase. Moving on to Section 3, we present and analyze the results from the screening phase, discuss the response surface design and its outcomes, and elaborate on the Mixed-Integer Nonlinear Multi-Objective Optimization model and its findings. Section 4 is dedicated to the validation of results, and finally, in Section 5, we offer a summary and conclusion for the paper.

## 2 Methodology

### 2.1 Specimen design and machine setup

**Fig. 2:**
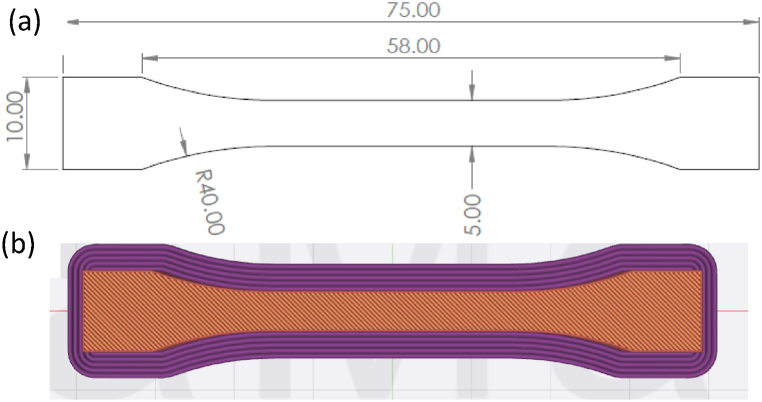
(a) A 2D drawing of the tensile specimen, and (b) Tensile Specimen with initial layer height of 0.3 mm and brim.

The testing specimen was designed using SolidWorks® with dimensions depicted in Fig. 2 (a). Subsequently, the 3D model was exported in stereolithography (STL) format to IdeaMaker® slicing software to configure the printing parameters. To ensure proper adhesion between the printed specimen and the build plate, the first layer of all samples was printed at a layer height of 0.3 mm, accompanied by a brim with parameters as illustrated in Fig. 2 (b).

The printing process occurred at room temperature. Each run commenced once the nozzle and plate temperatures simultaneously reached 25 °C. The Raise3D-E2 printer, featuring a 0.4 mm nozzle diameter, was utilized to fabricate specimens. A Polylactic Acid (PLA) filament material with a diameter of 1.75 mm was employed in this study, possessing a tensile strength of 46.6 MPa, modulus of elasticity of 2636 MPa, and a percent elongation of 1.9 %.

### 2.2 Mechanical strength and energy consumption measurements

**Fig. 3:**
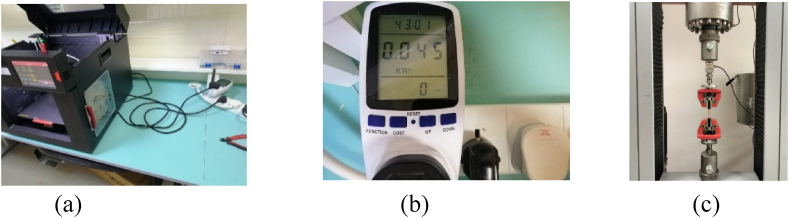
(a) 3D printer, (b) Energy meter, and (c) Tensile testing machine.

The 3D physical model was generated utilizing the Fused deposition modeling (FDM) 3D printing machine depicted in Fig. 3 (a). The energy consumption was monitored and measured using an energy meter with a precision of 0.001 KW-h. The energy meter was connected in-line between the printer and the power source. Once the printing process starts, the energy meter begins sampling the current and voltage levels of the supplied electricity to the printer. The sampled voltage and current levels are used to calculate instantaneous power consumption. The calculated power values over time are then integrated to determine the total energy consumed throughout the printing process, which is eventually shown on the energy meter display as in Fig. 3 (b). Tensile Strength assessments for each sample were conducted using the universal testing machine, Testometric FS300CT, equipped with a 2500 kg.f load cell, as indicated in Fig. 3 (c).

### 2.3 Design of experiment (DOE)

The majority of slicing software platforms provide a user interface for manipulating an extensive set of over 200 parameters relevant to 3D printers. However, the subset of parameters that significantly impact the operational processes of 3D printers is notably smaller. As depicted in Table 1, our investigation, supported by a comprehensive literature review [15,[39], [40], [41], [42], [43], [44], [45], [46], [47]] and our initial observations, has identified 8 key parameters believed to have a substantial influence on both mechanical characteristics and energy consumption. To systematically identify the most influential factors, we employed the Taguchi L16 orthogonal design, characterized by 2 levels and 4 replicates, for screening purposes.

**Table 1:** The suggested printing parameters and their respective levels.

Parameter	Symbol	Units	Levels	
			−1	1
Infill Percentage	A	%	40	80
Layer Height	B	mm	0.15	0.25
Number of Walls	C	–	1	4
Nozzle Temperature	D	°C	190	210
Printing Speed	E	mm/min	40	80
Bed Temperature	F	°C	30	60
Number of Bottom and Upper Layers	G	–	3	5
Infill Pattern	H	–	Honeycomb	Gyroid

## 3 Results

### 3.1 Screening results

Table 2 illustrates the mean Energy Consumption and the corresponding Tensile Strength values obtained from the Taguchi L16 orthogonal experimental design.

**Table 2:** The mean Energy Consumption and associated Tensile Strength metrics within the screening design.

	Factor Symbol									
No.	A	B	C	D	E	F	G	H	Energy Consumption (KWh)	Tensile Strength (MPa)
1	−1	−1	−1	−1	−1	−1	−1	−1	0.050	21.79
2	−1	−1	−1	1	−1	1	1	1	0.088	29.04
3	−1	−1	1	−1	1	−1	1	1	0.046	42.12
4	−1	−1	1	1	1	1	−1	−1	0.083	36.82
5	−1	1	−1	−1	1	1	−1	1	0.062	28.22
6	−1	1	−1	1	1	−1	1	−1	0.034	39.20
7	−1	1	1	−1	−1	1	1	−1	0.065	48.46
8	−1	1	1	1	−1	−1	−1	1	0.034	45.52
9	1	−1	−1	−1	1	1	1	−1	0.110	34.14
10	1	−1	−1	1	1	−1	−1	1	0.064	32.44
11	1	−1	1	−1	−1	1	−1	1	0.107	50.10
12	1	−1	1	1	−1	−1	1	−1	0.063	45.74
13	1	1	−1	−1	−1	−1	1	1	0.037	45.60
14	1	1	−1	1	−1	1	−1	−1	0.071	36.81
15	1	1	1	−1	1	−1	−1	−1	0.116	41.66
16	1	1	1	1	1	1	1	1	0.063	48.29

**Fig. 4:**
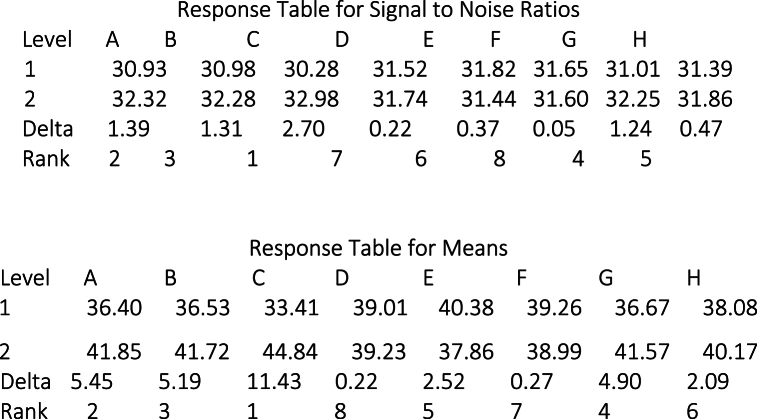
Taguchi analysis- tensile strength (Mpa) versus factors A, B, C, D, E, f, G, H.

Delta statistics for mean values and the signal-to-noise (S/N) ratio are utilized to identify the most influential factors from the set of eight proposed factors in Table 1. Fig. 4 illustrates the Delta statistics for Tensile Strength. The tabulated data distinctly indicate that Factors Number of Walls (C), Infill Percentage (A), Layer Height (B), and Bed Temperature (G) are the most consequential factors affecting Tensile Strength.

**Fig. 5:**
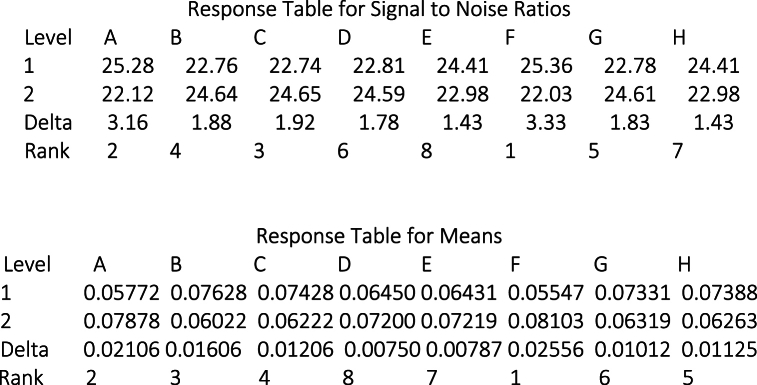
Taguchi analysis- energy Consumption (KWh) versus factors A, B, C, D, E, f, G, H.

Fig. 5 presents the Delta statistics related to Energy Consumption. The tabulated data distinctly show that Factor (F), representing the quantity of Bottom and Upper layers, (A) representing Infill Percentage, (C) representing the Number of Walls, and (B) representing Layer Height, are the most impactful factors influencing Energy Consumption in both mean and signal-to-noise ratio analyses.

Based on the observations illustrated in Fig. 4, Fig. 5, it is evident that Infill Percentage, Number of Walls, and Layer Height exert a clear influence on both Tensile Strength and Energy Consumption. Concerning the Number of Bottom and Upper Layers factor, it does indeed impact Energy Consumption, although its effect on Tensile Strength is insignificant (seventh rank). Conversely, Bed Temperature emerges as a significant factor influencing Tensile Strength and holds the fifth rank in terms of its impact on Energy Consumption. Consequently, we have chosen to focus on Infill Percentage, Number of Walls, Layer Height, and Bed Temperature as the key parameters for further in-depth investigation aimed at determining the optimal configuration that effectively balances the trade-off between Energy Consumption and Tensile Strength.

### 3.2 ANOVA

**Fig. 6:**
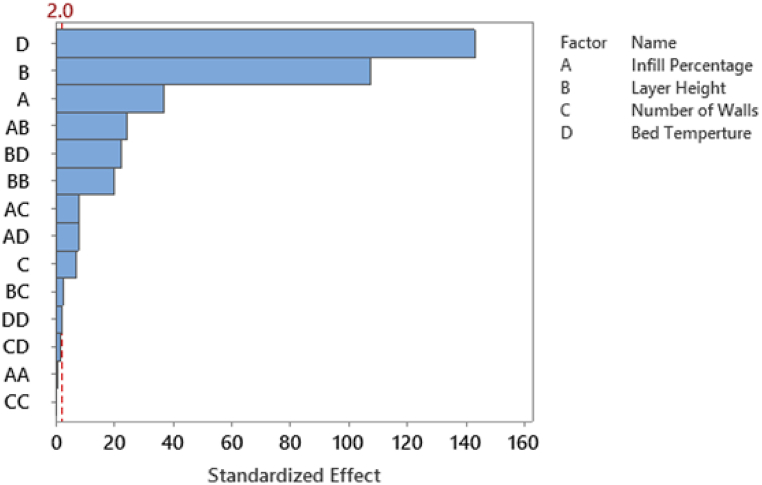
Standardized Effect for main and two-level interactions for Energy Consumption.

The chosen variables underwent a thorough analysis using Analysis of Variance (ANOVA) at a significance level α of 0.05, employing a linear model to determine their significance in relation to both Energy Consumption and Tensile Strength response variables. The results concerning Energy Consumption are detailed in Table 3 and visually depicted in Fig. 6.

**Table 3:** ANOVA results for main and two-level interactions for energy consumption.

Source	DF	Adj SS	Adj MS	F-Value	P-Value
Model	14	0.049	0.004	2572.000	0.000
Linear	4	0.046	0.011	8361.860	0.000
Infill Percentage	1	0.002	0.002	1357.760	0.000
Layer Height	1	0.016	0.016	11550.390	0.000
Number of Walls	1	0.000	0.000	48.360	0.000
Bed Temperature	1	0.028	0.028	20490.910	0.000
Square	4	0.002	0.000	330.720	0.000
Infill Percentage*Infill Percentage	1	0.000	0.000	0.570	0.451
Layer Height*Layer Height	1	0.001	0.001	409.310	0.000
Number of Walls*Number of Walls	1	0.000	0.000	0.030	0.865
Bed Temperature*Bed Temperature	1	0.000	0.000	4.090	0.045
2-Way Interaction	6	0.002	0.000	206.270	0.000
Infill Percentage*Layer Height	1	0.001	0.001	606.070	0.000
Infill Percentage*Number of Walls	1	0.000	0.000	63.210	0.000
Infill Percentage*Bed Temperature	1	0.000	0.000	60.200	0.000
Layer Height*Number of Walls	1	0.000	0.000	6.690	0.011
Layer Height*Bed Temperature	1	0.001	0.001	498.160	0.000
Number of Walls*Bed Temperature	1	0.000	0.000	3.310	0.071
Error	140	0.000	0.000		
Lack-of-Fit	10	0.000	0.000	27.850	0.000
Pure Error	130	0.000	0.000		
Total	154	0.049

**Fig. 7:**
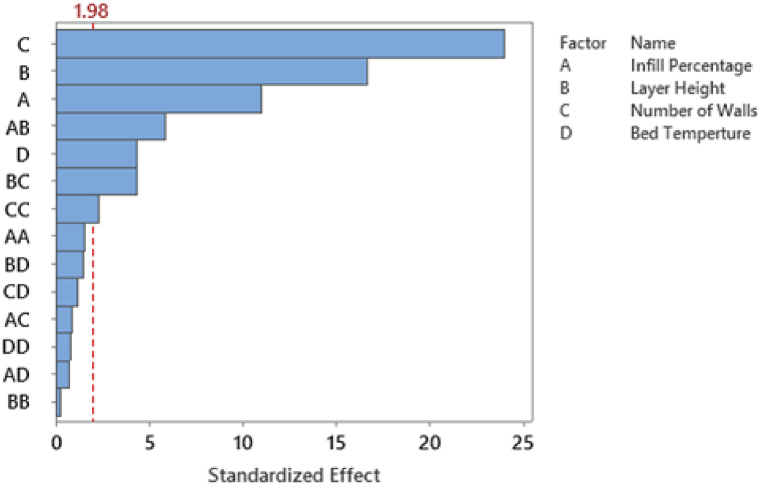
Standardized Effect for main and two-level interactions for Tensile Strength.

The p-values presented in Table 3 affirm the statistical significance of the four selected factors related to Energy Consumption. Additionally, the results indicate the importance of all two-way interactions involving these factors, except for the interaction between the Number of Walls and Bed Temperature, which fails to meet the 0.05 significance threshold. This discrepancy is further emphasized in the standardized effects figure depicted in Fig. 6. In this figure, it is evident that all main factors and their corresponding interactions demonstrate statistical significance, with the exception of the interaction between the Number of Walls and Bed Temperature. Table 4 and Fig. 7 showcase the outcomes for Tensile Strength at a significance level of α = 0.05.

**Table 4:** ANOVA results for main and two-level interactions for Tensile Strength.

Source	DF	Adj SS	Adj MS	F-Value	P-Value
Model	14	2068.410	147.740	76.790	0.000
Linear	4	1912.260	478.060	248.490	0.000
Infill Percentage	1	231.770	231.770	120.470	0.000
Layer Height	1	534.930	534.930	278.050	0.000
Number of Walls	1	1108.750	1108.750	576.310	0.000
Bed Temperature	1	36.810	36.810	19.130	0.000
Square	4	45.890	11.470	5.960	0.000
Infill Percentage*Infill Percentage	1	4.690	4.690	2.440	0.121
Layer Height*Layer Height	1	0.170	0.170	0.090	0.764
Number of Walls*Number of Walls	1	10.560	10.560	5.490	0.021
Bed Temperature*Bed Temperature	1	1.120	1.120	0.580	0.446
2-Way Interaction	6	110.260	18.380	9.550	0.000
Infill Percentage*Layer Height	1	65.270	65.270	33.930	0.000
Infill Percentage*Number of Walls	1	1.550	1.550	0.800	0.372
Infill Percentage*Bed Temperature	1	0.940	0.940	0.490	0.485
Layer Height*Number of Walls	1	35.740	35.740	18.580	0.000
Layer Height*Bed Temperature	1	4.020	4.020	2.090	0.150
Number of Walls*Bed Temperature	1	2.740	2.740	1.420	0.235
Error	140	269.340	1.920		
Lack-of-Fit	10	47.900	4.790	2.810	0.003
Pure Error	130	221.440	1.700		
Total	154	2337.750

The p-values presented in Table 4 affirm the significance of the selected four primary factors concerning Tensile Strength. Specifically, the analysis reveals that among these factors, only the interactions involving Infill Percentage*Layer Height and Layer Height*Number of Walls exhibit statistical significance at the α = 0.05 significance level. This observation is further substantiated by the Standardized Effect in Fig. 7, which distinctly illustrates the significance of all four primary factors. However, it emphasizes that only the interactions of Infill Percentage*Layer Height and Layer Height*Number of Walls remain statistically significant at the designated α = 0.05 significance level.

### 3.3 Face Centered Design Response Surface Analysis

A Face Centered Design (FCD) Response Surface analysis is employed to investigate the correlation between Tensile Strength and Energy Consumption concerning the four parameters derived from the screening analysis—namely Infill Rate, Layer Height, Number of Walls, and Bed Temperature. The FCD involves an experimental setup incorporating three levels for each of the aforementioned four parameters, as illuminated in Table 5.

**Table 5:** The printing parameters and their levels for FCD.

Parameter	Levels
Infill Percentage	40, 60, 80
Layer Height	0.15, 0.175, 0.25
Number of Walls	2, 3, 4
Bed Temperature	45, 55, 65

Table 6 exhibits the noteworthy significance of all primary factors in predicting Tensile Strength. Furthermore, the table shows the significance of the interactions involving Infill Percentage*Layer Height, as well as Layer Height*Number of Walls, at a significance level of α = 0.05. The adjusted R-squared stands at 91.4 %, signifying that 91.4 % of the variance observed in Tensile Strength is accounted for by the four primary factors and their respective interactions.

**Table 6:** Response surface regression: Tensile strength versus factors.

Estimated Regression Coefficients for Tensile Strength (Mpa)				
Term	Coef	SE Coef	T	P
Constant	44.0042	0.1630	269.982	0.000
Infill Percentage	1.6161	0.1235	13.089	0.000
Layer Height	2.5496	0.1234	20.657	0.000
Number of Walls	3.6320	**0.1264**	28.739	0.000
Bed Temperature	0.6480	0.1242	5.216	0.000
Infill Percentage*Infill Percentage	−0.6501	0.3324	−1.956	0.053
Layer Height*Layer Height	0.0670	0.3324	0.201	0.841
Number of Walls*Number of Walls	−0.4830	0.3837	−1.259	0.210
Bed Temperature*Bed Temperature	0.2200	0.3432	0.641	0.523
Infill Percentage*Layer Height	−0.8905	0.1312	−6.790	0.000
Infill Percentage*Number of Walls	−0.0134	0.1311	−0.102	0.919
Infill Percentage*Bed Temperature	0.2341	0.1311	1.785	0.077
Layer Height*Number of Walls	−0.6557	0.1312	−4.999	0.000
Layer Height*Bed Temperature	−0.2115	0.1312	−1.613	0.109
Number of Walls*Bed Temperature	**−0.0593**	0.1311	−0.452	0.652

S = 1.156 R-Sq = 92.3 % R-Sq(adj) = 91.4 %.

**Fig. 8:**
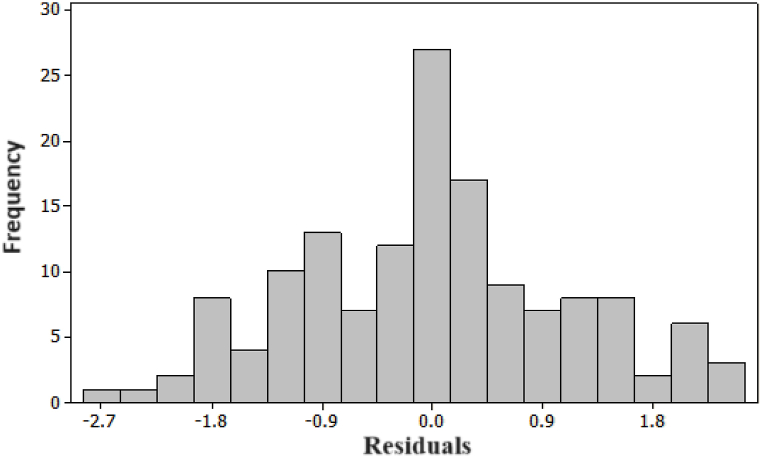
Histogram for the Tensile Strengths' residuals.

**Fig. 9:**
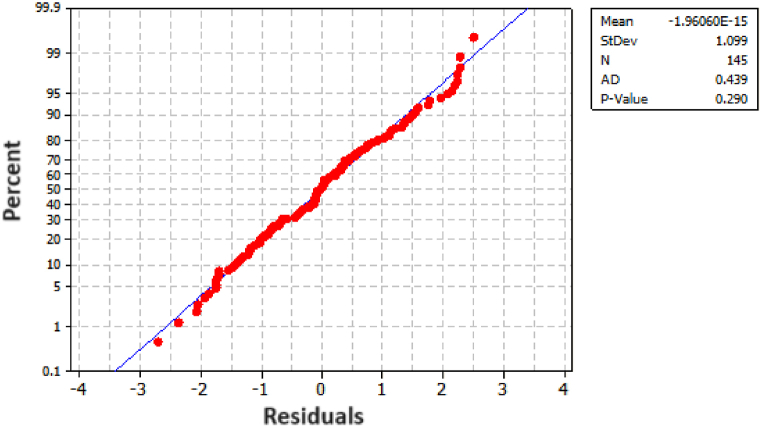
Probability Plots for Tensile Strength (MPa) residuals.

To assess the adequacy of the model, we constructed both a histogram and a normality plot for the residuals. As illustrated in Fig. 8, the histogram of the residuals demonstrates an approximate normal distribution. For a more quantitative analysis, Fig. 9 presents the probability plot for the residuals, revealing a p-value of 0.29. This p-value strongly suggests that the residuals follow a normal distribution, thereby affirming the adequacy of the model.

Utilizing the data presented in Table 6, Table 7 and assessing the model adequacy illustrated in Fig. 8, Fig. 9, we have formulated the regression equation that governs the relationship between Tensile Strength (TS) and the four primary factors, along with the consequential significant interactions. The equation is expressed as follows:

(1) Tensile Strength (TS) = 4.79221 + 0.317387Infill Percentage + 107.188*Layer Height + 8.42610*Number of Walls - 0.180232Bed Temperature - 0.593686Infill Percentage*Layer Height - 8.74205Layer Height*Number of Walls

**Table 7:** Estimated Regression Coefficients for Tensile Strength (Mpa) using data in.

Uncoded units	
Term	Coef
Constant	4.79221
Infill Percentage	0.317387
Layer Height	107.188
Number of Walls	8.42610
Bed Temperature	−0.180232
Infill Percentage*Infill Percentage	−0.00162536
Layer Height*Layer Height	11.9031
Number of Walls*Number of Walls	−0.482964
Bed Temperature*Bed Temperature	0.00219970
Infill Percentage*Layer Height	−0.593686
Infill Percentage*Number of Walls	−6.70736E-04
Infill Percentage*Bed Temperature	0.00117030
Layer Height*Number of Walls	−8.74205
Layer Height*Bed Temperature	−0.282038
Number of Walls*Bed Temperature	−0.00593147

Equation (1) reveals that higher values of Infill Percentage, Layer Height, and Number of Walls lead to an increase in Tensile Strength. Conversely, an increase in Temperature is associated with a decrease in Tensile Strength. Regarding the interaction effects at two levels, an elevated interaction between Infill Percentage and Layer Height corresponds to an increase in Tensile Strength, while heightened interaction between Layer Height and Number of Walls is associated with a decrease in Tensile Strength. In conclusion, the influence of individual factors on Tensile Strength depends on their values and interactions, lacking a clear, consistent direction for the relationship between any factor and Tensile Strength.

Table 8 demonstrates the statistical significance of all primary factors and their interactions in predicting energy consumption, excluding the second-order Infill Percentage and Number of Walls. This determination is made at a significance level of α = 0.05. The adjusted R-squared value stands at an impressive 99.6 %, signifying that nearly the entire variability (99.6 %) in energy consumption is accounted for by the four primary factors and their interactions (see Table 9).

**Table 8:** Response surface regression: Energy consumption versus factors.

Estimated Regression Coefficients for Energy Consumption (KWh)				
Term	Coef	SE Coef	T	P
Constant	0.056098	0.000155	362.294	0.000
Infill Percentage	0.004533	0.000123	36.848	0.000
Layer Height	−0.013222	0.000123	−107.473	0.000
Number of Walls	−0.000856	0.000123	−6.954	0.000
Bed Temperature	0.017611	0.000123	143.146	0.000
Infill Percentage*Infill Percentage	−0.000245	0.000324	−0.756	0.451
Layer Height*Layer Height	0.006555	0.000324	20.231	0.000
Number of Walls*Number of Walls	0.000055	0.000324	0.170	0.865
Bed Temperature*Bed Temperature	0.000655	0.000324	2.022	0.045
Infill Percentage*Layer Height	−0.003212	0.000130	−24.618	0.000
Infill Percentage*Number of Walls	−0.001038	0.000130	−7.951	0.000
Infill Percentage*Bed Temperature	0.001013	0.000130	7.759	0.000
Layer Height*Number of Walls	0.000337	0.000130	2.586	0.011
Layer Height*Bed Temperature	−0.002913	0.000130	−22.319	0.000
Number of Walls*Bed Temperature	−0.000238	0.000120	−1.930	0.049

S = 0.001167 R-Sq = 99.6 % R-Sq(adj) = 99.6 %.

**Table 9:** Estimated Regression Coefficient for Energy Consumption

Estimated Regression Coefficients for Energy Consumption (KWh) using data in uncoded units	
Term	Coef
Constant	−0.0259431
Infill Percentage	0.000552086
Layer Height	−0.255592
Number of Walls	0.00244449
Bed Temperature	0.00148747
Infill Percentage*Infill Percentage	−6.12000E-07
Layer Height*Layer Height	1.16537
Number of Walls*Number of Walls	5.52000E-05
Bed Temperature*Bed Temperature	6.55200E-06
Infill Percentage*Layer Height	−0.00214167
Infill Percentage*Number of Walls	−5.18750E-05
Infill Percentage*Bed Temperature	5.06250E-06
Layer Height*Number of Walls	0.00450000
Layer Height*Bed Temperature	−0.00389333
Number of Walls*Bed Temperature	−2.37500E-05

**Fig. 10:**
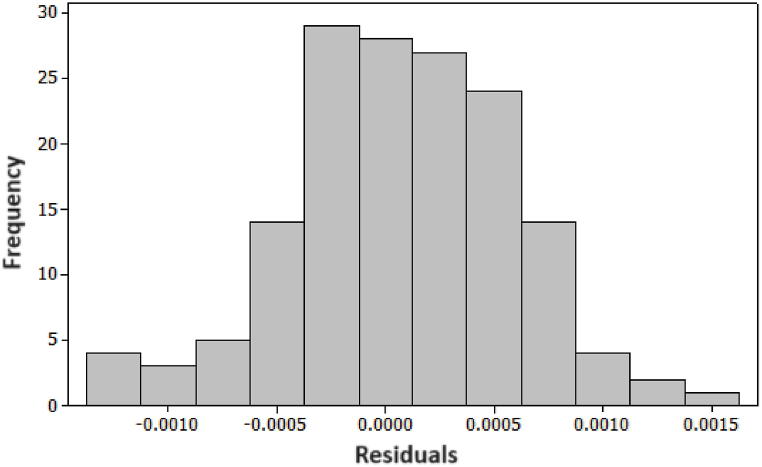
Histogram for the Energy Consumption's residuals.

**Fig. 11:**
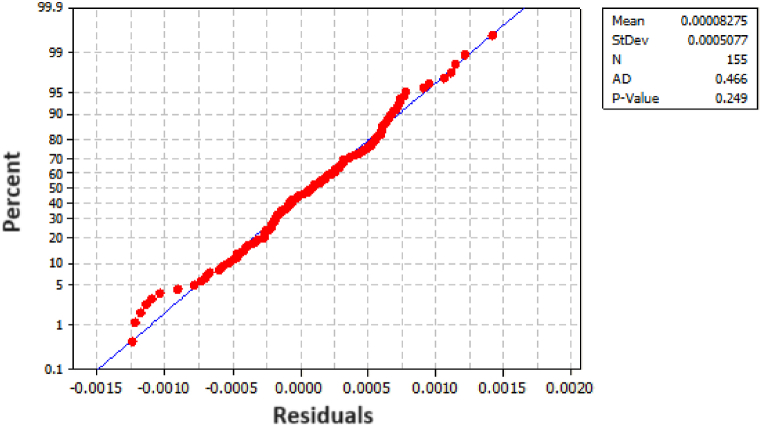
Probability plots for energy Consumption (KWh).

To verify model's adequacy, we constructed both a histogram and a normality plot for the residuals. As depicted in Fig. 10, the histogram of the residuals illustrates an approximate normal distribution. In pursuit of a more quantitative analysis, Fig. 11 showcases a probability plot for the residuals, revealing a p-value of 0.249. This value strongly suggests that the residuals conform to a normal distribution, affirming the adequacy of the model.

Based on the data presented in Table 8, Table 9, and considering the model adequacy illustrated in Fig. 10, Fig. 11, the regression equation linking Tensile Strength to the primary factors and their significant interactions can be formulated as follows:

(2) Energy Consumption (EC) = -0.0259431 + 0.000552086Infill Percentage - 0.255592Layer Height + 0.00244449Number of Walls + 0.001487472Bed Temperature + 1.16537(Layer Height)^2 + 6.55200*10^-6 (Bed Temperature)2–0.00214167Infill Percentage*Layer Height - 5.18750*10-5Infill Percentage*Number of Walls + 5.06250*10-6*Infill Percentage*Bed Temperature + 0.00450000Layer Height*Number of Walls - 0.00398333Layer Height*Bed Temperature

Equation (2) demonstrates that elevating Infill Percentage, Number of Walls, and Bed Temperature leads to an increase in Energy Consumption. The impact of Layer Height on energy consumption is less straightforward, exhibiting a positive linear relationship and a negative second-order relationship. Regarding interaction effects at two levels, heightened interaction between Infill Percentage and Temperature, as well as Layer Height and Number of Walls, corresponds to an increase in Energy Consumption. Conversely, increased interaction between Infill Percentage and Layer Height, Infill Percentage and Number of Walls, and Layer Height and Bed Temperature is linked to a decrease in Energy Consumption. In conclusion, the influence of individual factors on Energy Consumption depends on their values and interactions. There's no clear, consistent pattern for the relationship between any single factor and Energy Consumption.

### 3.4 Mixed-Integer Nonlinear Multi-objective optimization

The Mixed-Integer Nonlinear Multi-Objective optimization model is designed to minimize Energy Consumption EC while adhering to the lower limit Tensile Strength TS constraint of 50 MPa. Equation (3) defines this optimization model:

(3) Min EC s.t. TS ≥ 50 50 ≤ Infill Percentage ≤ 90 0.1 ≤ Layer Height ≤ 0.3 Number of Walls ∈{1,2,3,4} 40 ≤ Bed Temperature ≤ 70,

The optimization problem was solved using Python's SciPy library, specifically the optimize linprog function. The resultant optimal parameters have been presented in Table 10.

**Table 10:** Optimal parameters.

Parameter	Optimal Value
Infill Percentage	90
Layer Height	0.3
Number of Walls	4
Bed Temperature	60

**Fig. 12:**
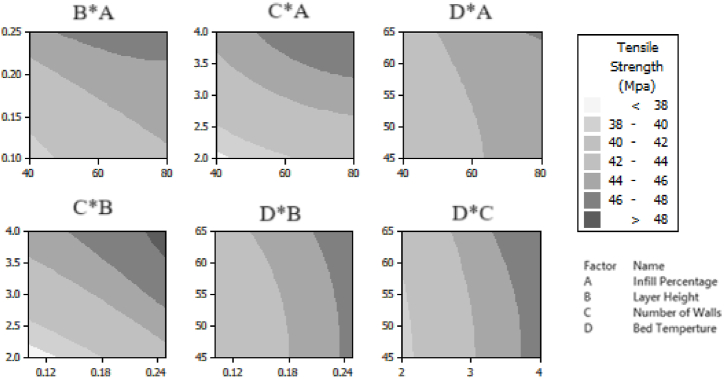
Contour plots for tensile strength.

The contour plots generated by Response Surface analysis in Fig. 12 confirm what we found in Table 10. When we look at the graph for Bed Temperature and Infill Percentage, we can see that Tensile Strength is at its highest when the Bed Temperature is higher than 63 °C and the Infill Percentage is above 77 %. Another graph, showing the Number of Walls and Layer Height, indicates that Tensile Strength is highest when the Number of Walls is more than 3.5 and the Layer Height is over 0.22 mm. As Table 10 presents, our optimization model suggests an Infill Percentage of 90 %, a Layer Height of 0.3 mm, a Number of Walls of 4, and a Bed Temperature of 60 °C, aligning with what the contour plots suggest except for Bed Temperature. Notably, the optimization model recommends a Bed Temperature of 60 °C, which is lower than what the contour plot suggests. This decision is based on minimizing Energy Consumption, which is directly related to Bed Temperature, as shown in Equation (2). It's evident that the Optimization Model compromises by finding a solution that balances between the two conflicting objectives: maximizing Tensile Strength and minimizing Energy Consumption.

## 4 Validation

To validate these parameters, a set of five specimens were fabricated employing the specified values. Subsequently, their Tensile Strengths and Energy Consumptions were measured and compared against the predicted values computed via Equations (1), (2)). The tabulated data illustrating these outcomes is presented in Table 11.

**Table 11:** Predicted versus actual TS and EC.

No.	Actual TS (Mpa)	Predicted TS (Mpa)	Prediction Error%	Actual EC (KWh)	Predicted EC (KWh)	Prediction error%
1	51.852	49.5	4.5 %	0.053	0.0529	0.19 %
2	50.369		1.7 %	0.052		−1.73 %
3	51.011		3.0 %	0.052		−1.73 %
4	51.311		3.5 %	0.052		−1.73 %
5	51.221		3.4 %	0.052		−1.73 %

The percentage error for Tensile Strength is observed to be below 5 %, while the error percentage for Energy Consumption is less than 2 %. This clearly signifies that the Tensile Strength and Energy Consumption are substantially influenced by the factors chosen in the screening phase, namely, Infill Percentage, Layer Height, Number of walls, and Bed Temperature. Consequently, an accurate prediction of the Tensile Strength and the Energy Consumption of the specimen made by the 3D printer can be achieved by precise knowledge of the Infill Percentage, Layer Height, Number of walls, and Bed Temperature values.

## 5 Conclusion

In summary, this study effectively employed a comprehensive methodology to optimize critical parameters of a Fused Deposition Modeling (FDM) 3D printer, with a focus on minimizing Energy Consumption (EC) while surpassing a predefined lower threshold for Tensile Strength (TS). The investigation utilized a Design of Experiments (DoE) approach, integrating Taguchi analysis, Response Surface Analysis, and Mixed-Integer Nonlinear Multi-Objective Optimization to assess the impact of key printing parameters (Infill Percentage, Layer Height, Number of Walls, and Bed Temperature) on both TS and EC.

The Taguchi screening analysis identified Infill Percentage, Layer Height, Number of Walls, and Bed Temperature as the most influential factors affecting TS and EC. Response Surface Analysis generated regression models for TS and EC, enabling precise predictions based on the chosen parameters. The subsequent Mixed-Integer Nonlinear Multi-Objective Optimization successfully balanced TS and EC, minimizing EC while maintaining a specified lower limit for TS.

The validation phase, involving the fabrication of specimens with optimal parameter values, demonstrated the efficacy of the proposed methodology. The observed percentage error for Tensile Strength remained below 5 %, while the error percentage for Energy Consumption was less than 2 %. These results underscore the significant role of Infill Percentage, Layer Height, Number of Walls, and Bed Temperature in determining both TS and EC in FDM 3D printers.

This study's findings offer valuable insights for optimizing FDM 3D printing processes, particularly in achieving a trade-off between energy efficiency and mechanical strength. The proposed methodology not only enhances the understanding of the interplay between key parameters but also provides a practical approach for achieving desired outcomes in 3D printing applications. Future research may explore additional parameters and advanced optimization techniques to further refine the process and broaden its applicability across diverse manufacturing scenarios.

## Data availability

The data associated with this study has not been deposited into a publicly repository. However, the data underlying this study are available upon request. Please contact Saleem Ramadan at saleem.ramadan@htu.edu.jo for access to the data."

## CRediT authorship contribution statement

**Saleem Ramadan:** Writing – review & editing, Visualization, Validation, Software, Resources, Investigation, Formal analysis, Conceptualization. **Qutaiba Altwarah:** Validation, Methodology, Investigation. **Mohammad Abu-Shams:** Writing – original draft, Visualization, Validation, Conceptualization. **Duha Alkurdi:** Visualization, Validation, Conceptualization.

## Declaration of competing interest

The authors declare that they have no known competing financial interests or personal relationships that could have appeared to influence the work reported in this paper.
